# Coiling of echogenic perineural catheters with integral stylet: A proof-of-concept randomized control trial in a sciatic nerve block simulator and a pilot study in orthopaedic-trauma patients.

**DOI:** 10.12688/f1000research.155381.1

**Published:** 2024-09-27

**Authors:** Theodosios Saranteas, Eleni Poulogiannopoulou, Maria Riga, Konstantina Panagouli, Andreas Mavrogenis, Thomas Papadimos

**Affiliations:** 1 Second Department of Anaesthesiology, National and Kapodistrian University of Athens School of Medicine, Athens, Attica, 15349, Greece; 2 First Department of Orthopaedics, National and Kapodistrian University of Athens School of Medicine, Athens, Attica, 15349, Greece; 3 Department of Anaesthesiology, University of Toledo College of Medicine and Life Sciences, Toledo, Ohio, 43614, USA

**Keywords:** perineural catheter coiling; sciatic nerve block; ultrasound; catheter tip dislocation; simulation.

## Abstract

**Backround/Objectives:**

We investigated a technique that facilitates the coiling of a regular straight catheter (with integral stylet) behind the sciatic nerve in an ultrasound (US) regional anaesthesia simulator, and then applied our findings to a series of orthopedic-trauma patients.

**Methods:**

We conducted a randomized study of two methods of perineural catheter advancement in a sciatic nerve block Blue Phantom simulator. Two groups of twenty catheters each (method A and method B) were evaluated under real-time ultrasound imaging. The needle in-plane/nerve in-short-axis technique was applied. In method A the catheter was advanced beyond the needle tip with the integral stylet extending along its entire length; in method B the catheter was advanced after its integral stylet was retracted by 6 cm, thus providing flexibility to the catheter’s distal end. Additionally, to assess the procedural effectiveness of method B coiling technique, a pilot study was conducted examining 25 perineural catheters coiled underneath the sciatic nerve in trauma-orthopaedic patients to document any displacement of catheters’ tip from their initial position (for 36 hours postoperatively).

**Results:**

In the simulation study, method B led to a significantly higher percentage (18/20:90%) of coiled catheters than method A (3/20:15%). Two coiled catheters of method B were found kinked/obstructed. In our patients, after catheter insertion, the distal end of 2/25 (8%) coiled catheters was obstructed. One perineural catheter was dislodged. For the remaining 22 (88%) catheters, ultrasound imaging demonstrated that local anaesthetic infusion made contact with the sciatic nerve, indicating no displacement of the catheter’s distal end postoperatively.

**Conclusion:**

Regular straight perineural catheters can be easily coiled if their integral stylet is partially retracted. This coiling method offers extra catheter length adjacent to the nerve structure which potentially mitigates catheter tip displacement.

**Trial registration:**

clinicaltrials.gov, registration No: NCT06568510, 23/08/2024, registration URL: https://clinicaltrials.gov/study/NCT06568510?intr=coiling%20of%20echogenic%20sciatic%20nerve&rank=1#study-overview

## Trial registration

clinicaltrials.gov, registration No: NCT06568510, 23/08/2024, registration URL: https://clinicaltrials.gov/study/NCT06568510?intr=coiling%20of%20echogenic%20sciatic%20nerve&rank=1#study-overview

## Introduction

Self-coiling catheters and catheters with a flexible, distal end-portion and wire-reinforced body can be looped, providing additional catheter length adjacent to the targeted nerves. Coiling potentially lowers dislocation rates of continuous peripheral nerve block (CPNB) catheters. ^1^^,^^2^

Regular straight perineural catheters inserted with the needle in-plane/nerve in-short-axis technique, tend to bypass the nerve due to the perpendicular orientation of the needle/catheter relative to the nerve. ^3^^,^^4^ As of this time, there are no investigations examining whether regular straight perineural catheters (inserted with the needle in-plane/nerve in-short-axis technique) can be looped adjacent to nerves, and consequently, mitigate CPNB failure secondary to displacement.

Therefore, we investigated a technique that facilitates the coiling of a regular straight catheter (with integral stylet) behind the sciatic nerve in an ultrasound (US) regional anaesthesia simulator, and then applied our findings to a series of orthopedic-trauma patients.

## Methods

### Study design and settings

Two independent studies were conducted: 1) A simulation-based protocol in a Blue Phantom US simulator of sciatic nerve block. 2) A pilot study in 25 patients undergoing sciatic CPNB. All procedures were performed under US real-time visualization (5-9 MHz linear transducer; LOGIQ e; GE Healthcare, USA).

### Simulation-based CPNBs methods and randomization

Two methods of perineural catheter advancement using the Blue Phantom simulator were evaluated. A 20G echogenic straight perineural catheter (SonoLong Sono, PAJUNK, Germany) with a stainless-steel helical coil-reinforced body and a steel integral stylet was used.

**Figure 1.**
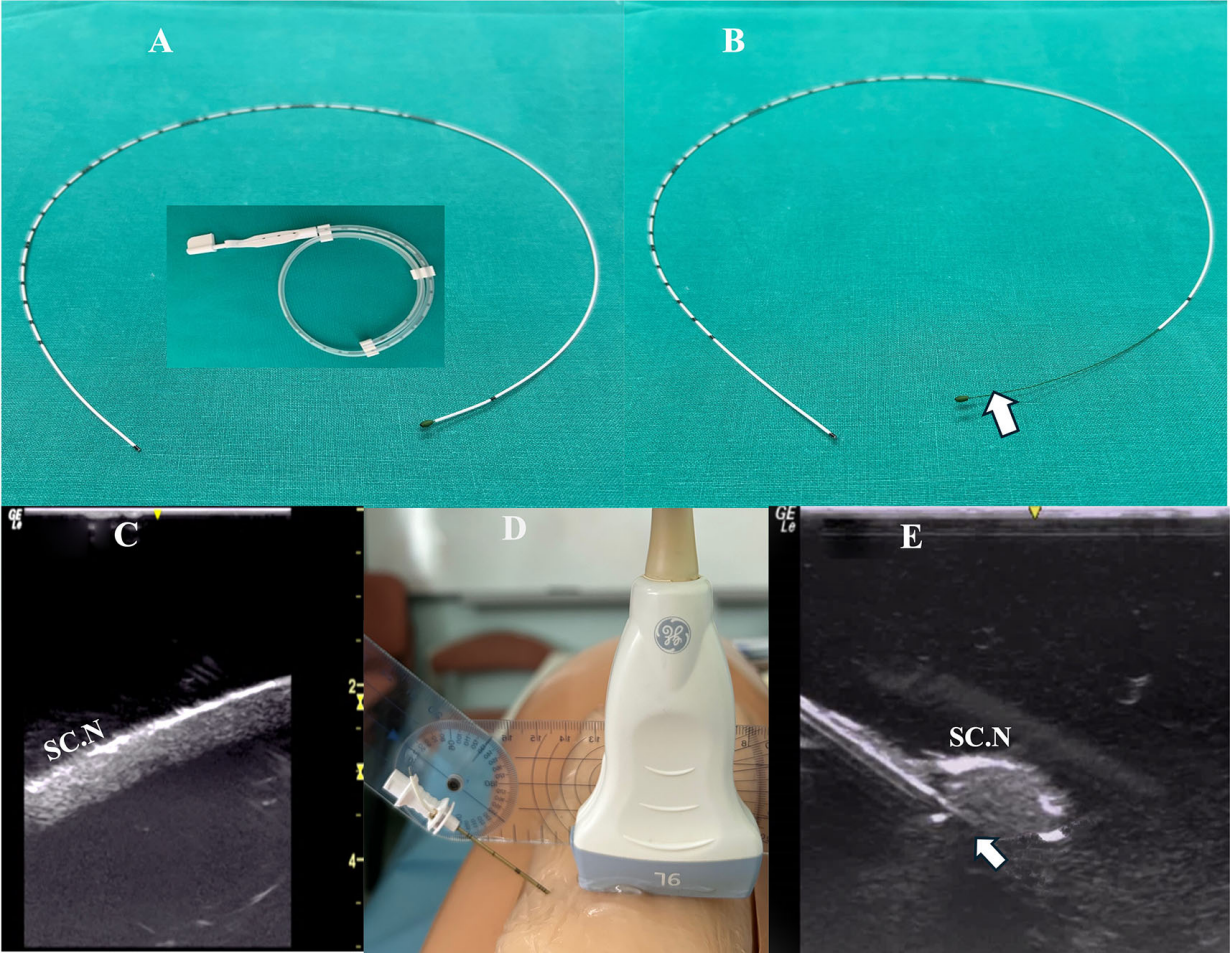
A: The perineural catheter removed from catheter’s container, with the integral stylet extending along its entire length (from the beginning to the tip); B: The catheter’s integral stylet is retracted by 6 cm (arrow) so that the catheter’s distal portion features a more flexible end; C: Blue Phantom’s sciatic nerve in long axis view; D: the needle insertion angle (40 degrees) to the Blue Phantom posterior thigh surface as measured by goniometer; E: Ultrasound image of the Tuohy needle tip (arrow) as directed underneath the sciatic nerve of the simulator. **Inset: perineural catheter container.** SC.N=Sciatic Nerve.

Method A: The catheter was advanced beyond the needle tip (under real-time US imaging) with its integral stylet extending along the entire length of the catheter ( Figure 1A).

Method B: The catheter was advanced beyond the needle tip after the catheter’s integral stylet was retracted by 6 cm so that the catheter’s distal portion provided a more flexible end ( Figure 1B).

Randomization was done using a randomization generator ( www.randomization.com) by means of sequentially numbered sealed opaque envelopes, including information about the sequence of method allocation.

The simulator was provided with a sciatic nerve insert (upper third of the posterior thigh) containing one nerve structure (CAE training Blue Phantom). The sciatic nerve-simulator surface distance featured different location depths (2-4 cm) ( Figure 1C).

**Figure 2.**
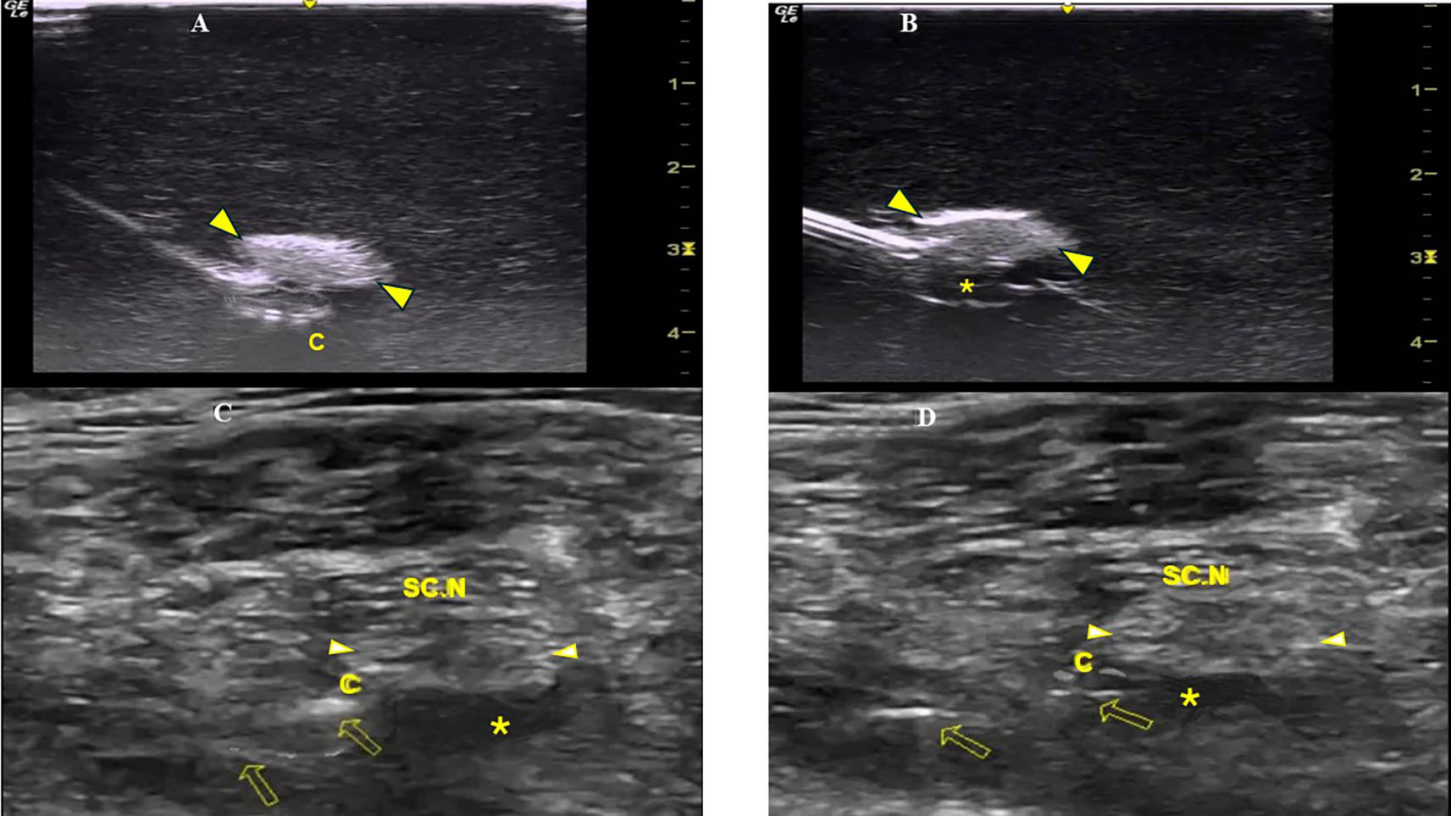
A: The coiled perineural catheter underneath the sciatic nerve (arrowheads) of the Blue Phantom simulator; B: Saline spread contacts the sciatic nerve (arrowheads) of the Blue Phantom simulator; C: The injection of LA through the perineural catheter (arrows) contacts the pantient’s sciatic nerve (arrowheads) prior to surgery; and D: 36 hours postoperatively. C=Perineural Catheter, SC.N=Sciatic Nerve, *=LA spread.

A Tuohy needle (18G × 7.5 mm, PAJUNK, Germany) was inserted at the simulator’s posterior thigh (TS) from a lateral to medial direction (needle in-plane/nerve in-short-axis technique). The needle insertion angles (coronal plane) were always kept at 40 degrees ( Figure 1D). In both methods, the needle was aimed behind the sciatic nerve with the curved end facing the nerve ( Figure 1E). The perineural catheter was threaded through the Tuohy needle until its tip reached the needle orifice. The catheter was slowly advanced exactly 6 cm (under real-time US guidance) beyond the curved tip of the Tuohy needle, until the catheter’s distal end-portion coiled exactly under the sciatic nerve ( Figure 2A). To better visualize a catheter’s trajectory, a slight heel-toe maneuver was implemented to bring the US beam perpendicular to catheter’s distal portion. Procedural time was limited to 15 seconds (total allowed performance time for each coiling maneuver). Each attempt was stored digitally and analyzed off-line (frame by frame) by two co-investigators (EP, MR) who were blinded to group allocation. The two investigators decided independently whether the catheter coiled or not. The result was considered positive (coiled catheter) only after unanimous agreement between the two investigators. If the catheter distal end did not coil or if the catheter bypassed the nerve and then coiled, the catheter was withdrawn, the needle tip reoriented (rotation of needle shaft/tip at 90 degrees), and the catheter was readvanced. In total, three attempts were allowed (overall maximum procedural time: 45 seconds). The number of attempts to coil a catheter, as well as the proportion of cases where a catheter was successfully looped on first attempt, were measured. The patency of coiled catheters was tested after normal saline (3 ml) infusion through the catheter. Contact of saline spread with the nerve was also recorded ( Figure 2B). All images were taken under the same imaging settings (gain: 90 db, focus position at the level of the nerve, standard time gain compensation).

### Statistical analysis

Sample size calculation was based on a previous pilot simulation study including 10 CPNBs for each method, using a Blue Phantom nerve block simulator that contained two nerve structures with dimensions of 17cm × 13cm × 6cm (L × H × W), (CAE-Healthcare). Catheters were coiled (after maximum of three attempts) in 3/10 (30%) and in 8/10 (80%) cases, with method A and B respectively. Using an α error of 0.025 and a power of (1− β) at 80% to detect a 50% difference between the two methods, a sample size of 18 CPNBs in each group was required. To allow for increased data variability, 40 CPNBs were performed in a sciatic nerve block simulator. Proportions were compared using Fisher’s exact test; *p* values <0.05 were considered statistically significant. Interobserver agreement was assessed by using the inter-rater agreement statistic Kappa (MedCalc Software, Mariakerke, Belgium).

### Pilot study in orthopaedic-trauma patients

To assess procedural effectiveness of the preferred coiling technique (method B), a pilot study was performed. The pilot study was conducted according to the principles expressed in the Declaration of Helsinki and was approved by the “Attikon” Hospital Ethics Committee (Approval No 342, Approval Date 09/05/2024). Informed written consent was always obtained from the patients or their surrogates.

Straight perineural catheters with coiled distal ends behind the sciatic nerve were examined in 25 patients (age: 28-55 years; male/female: 14/11; BMI: 26 (19-31) kg/m ^2^) who had sustained tibia fractures (recruitment period: 10/05/2024-15/07/2024). Exclusion criteria: patients with infection in the subgluteal region, allergies to local anaesthetics, patients’ refusal to consent for the nerve block, neurological disorders, pregnancy.

Patients were placed in the lateral position (with the injured leg uppermost) and a US-guided sciatic nerve block was performed under aseptic conditions (TS, EP). The sciatic nerve was visualized at the upper third of the posterior thigh (short axis view) and a Tuohy needle (21 G × 10 mm, SonoLong/NanoLine, PAJUNK, Germany) was inserted in a lateral to medial direction. The needle was placed underneath the sciatic nerve and 10 ml of ropivacaine (0.1%) were injected. A perineural catheter (with retracted integral stylet by 6 cm) (SonoLong Sono, PAJUNK, Germany) was then threaded through the needle tip and coiled behind the sciatic nerve. Confirmation of correct catheter tip placement was defined as adequate spread and contact of ropivacaine injectate (3 ml, 0.1%) with the sciatic nerve ( Figure 2C). If the distribution of the local anaesthetic (LA) could not be visualized on the first infusion, 2 additional injections were performed until the distribution was clearly seen. If LA spread could not be visually confirmed, or if, when confirmed, LA did not come in contact with the sciatic nerve, the case was excluded.

Subcutaneous tunneling (4 cm long) and placement of a transparent adhesive dressing were used to secure the catheter. All sciatic nerve blocks were followed by an adductor canal block (10 ml of ropivacaine 0.5%) and general anaesthesia. A continuous infusion 8-12 ml of ropivacaine 0,1% (ROPIVACAINE/KABI INJ.SOL 2 mg/ml, 28823.01.08, Fresenius Kabi Hellas A.E.) was commenced and an oral combination of paracetamol 325 mg with oxycodone 5 mg (DEPALGOS F.C. TAB 325+5 mg, 91337.01.01, MOLTENI SPA, Italy) was provided on an as-needed basis after surgery.

All CPNBs were assessed and data were collected in the post-anaesthetic care unit, and every 12 hours thereafter until 36 hours postoperatively from members of the acute pain service (APS) team not participating in the study. Observation of fluid under the dressing indicated catheter’s leakage. Unplanned external displacement and dislodgement of the catheter were defined: less than and equal to 6 cm (displacement) and more than 6 cm (dislodgement) movement of the catheter from the initial recorded depth at the point of insertion or from the distal end of the subcutaneous tunnel. The final catheter tip position was evaluated (under US imaging) 36 hours postoperatively and was identified by injecting 3 ml of ropivacaine (0.1%) through the catheter ( Figure 2D). If LA spread was not confirmed after 3 injections or if, when confirmed, it did not come in contact with the sciatic nerve, the catheter’s tip was considered dislocated.

## Results

**Figure 3.**
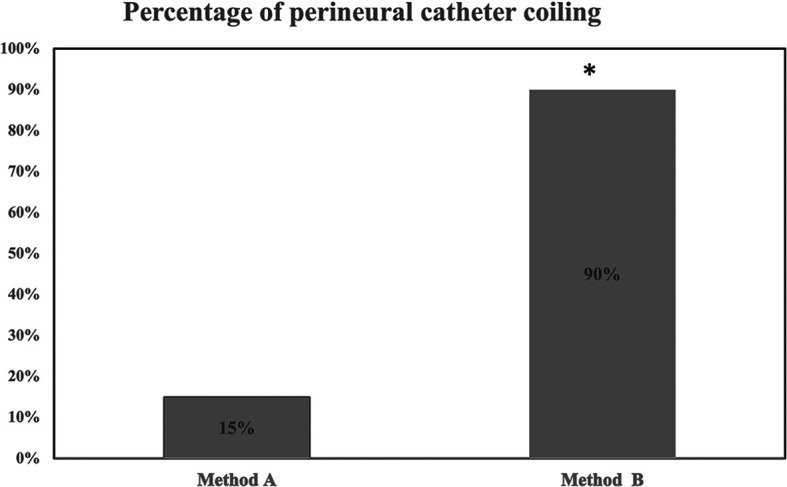
Percentage of coiling catheters in method A and method B (catheter’s end portion stylet unsupported). * denotes *p*=0.0003 (method A vs method B).

In the simulation study, overall 21 catheters coiled behind the sciatic nerve in both groups. None of the catheters coiled after bypassing the nerve. Removal of integral stylet (group B) led to a higher percentage (18/20:90%) of coiling catheters than in group A (3/20:15%), *p*=0.0003 ( Figure 3). Of group A (n=3) and group B (n=18) coiled catheters, 3/3 (100%) and 15/18 (83%) ( *p*=0.9) coiled on first attempt, respectively. There was significant agreement (kappa=0.9) between the two investigators assessing perineural catheter coiling. In 2 out of 18 coiled catheters (group B), saline injection was not feasible due to catheter’s distal end kinking/obstruction. Saline injections contacted the nerve in 3/3 (100%) (group A) and in 13/16 (81%) (group B) coiled catheters, respectively. After gradual uncoiling of the 2 obstructed catheters by 1 and 2 cm respectively, successful injection of saline became possible and made contact with the nerve. ^5^

In sciatic nerve CPNBs in patients with tibia fractures, the distal end of 2/25 (8%) coiled catheters was kinked/obstructed after catheter insertion, but after uncoiling the catheters by 3 cm and 2 cm, ropivacaine administration was successful. One catheter was dislodged postoperatively. Of the 22 indwelling catheters evaluated 36 hours postoperatively, the LA spread was visualized in all US examinations. Although 5/22 (22%) catheters were found externally displaced (displacement length: 2 (2-3) cm) postoperatively, no catheter tips were found dislocated. No leakage around the catheters was noticed. Neither catheter knotting nor nerve trapping/injury were recorded. Oxycodone consumprion was 12.5 (0-20) mg and the average numeric rating scale (NRS) score: 2 (0-5). ^6^

## Discussion

Assessment of a new technique ideally should take place in humans. However, the methodology to sufficiently evaluate needles and perineural catheters on patients presents difficulties and/or ethical concerns. ^7^^–^^11^ Therefore, we decided to initially assess this specific perineural catheter method in a “human-tissue mimicking” simulation model.

The Blue Phantom simulator was selected because its physical properties are close to human tissue and also has been employed as a testing method in peripheral nerve block needle visibility research endavours. ^12^^–^^17^ Additionally, the Blue Phantom sciatic nerve block simulator exhibits an anatomic configuration approximating that of human anatomy.

Furthermore, by partially withdrawing the integral stylet, the catheter body remained resistant to bending, while the catheter’s distal end developed a balance of malleability and strength that was required to achieve an ease of coiling, as well as lower probability for inadvertent penetration and nerve injury. We also used a Tuohy needle because its curved tip facilitated a trajectory (directional curvature) of the catheter that allowed proper target placement.

In our simulation study, the needle-insertion angles were maintained constant to attain standard angulations between the catheter’s tip and the surrounding Blue Phantom material once the catheter was threaded out of the Tuohy needle. We did not use shallow 0 to 30 degrees catheter insertion angles to optimize echogenicity because the Blue Phantom’s material is homogenous with low background echogenicity and enhances needle (and consequently perineural catheter) visibility, especially at low needle insertion angles. ^7^ We decided, thus, to implement a steeper insertion angle of 40 degrees to simulate more difficult clinical conditions. Nevertheless, in all cases, the operator could easily advance the catheter and the two independent investigators could visually assess the trajectory of the catheters with precision and significant interobserver agreement.

In our patients, US examination of the perineural catheters (36 hours postoperatively) did not reveal any catheter tip dislocation (internally migrated catheter tip) from their initial position placement (underneath the sciatic nerve); even though 22% (5/22) of them exhibited external displacement at the point of insertion or from the distal end of the subcutaneous tunnel. It has been previously reported that regular straight perineural catheters with integral stylet display a lower tip dislocation rate when placed with the needle out-of-plane/nerve in-short axis approach. ^4^^,^^18^ However, we can advocate that this type of perineural catheter could alternatively be inserted with good results, i.e. low dislocation rates of catheter’s tip, if the needle in-plane/nerve in-short axis approach is combined with the coiling CPNB technique.

Leakage of perineural catheters is a shortcoming of the catheter-through-needle method. ^19^ In our case series, no perineural leakage was recorded. It could be considered that a 6 cm coiled catheter path in perineural tissues may be long and winding enough to prevent local anesthetic from flowing back through the needle-puncture track. However, further investigation is imperative to support this hypothesis.

A procedural weakness is the kinking of the distal end of the coiled catheters. Nevertheless, by partially uncoiling the catheter, points of obstruction can be relieved. We based our 6 cm stylet retraction on a previous report which although using a different perineural catheter brand, had a fixed 6 cm flexible/soft distal end as well. ^2^ Although an advantage of this technique is that the stylet can be retracted from the catheter’s tip at different lengths, we did not study various lengths of stylet withdrawal (and consequently the optimal length affecting a catheter’s flexible end) that could facilitate the coiling maneuver without obstruction of the catheter.

Moreover, attention should be paid to if extrapolating these observations to other ultrasound approaches, perineural catheter placement methods, as well as to diverse time-intervals of LA infusions.

## Conclusion

A regular straight perineural catheter can be easily coiled if its integral stylet is partially retracted. This coiling method secures a safe and adequate extra length that delivers to the operator a margin of comfort regarding the mitigation of perineural catheter tip dislocation. Further studies are required to evaluate the clinical effectiveness of this perineural catheter coiling method over other catheters’ designs and techniques.

### Ethics and consent

The pilot study was conducted according to the principles expressed in the Declaration of Helsinki and was approved by the “Attikon” Hospital Ethics Committee (Approval No 342, Approval Date 09/05/2024). In accordance with the “Attikon” Hospital Ethics Committee requirements, informed written consent was always obtained from the patients or their surrogates.

## Author contributions

TS: conception and implementation of the technique, writing, and correcting the manuscript. EP: equal contribution to the first author at all stages of the study. MR and KP: data collection and analysis. AM: recruiment and follow-up of patients. TP: commenting on all stages of the study and drafting the manuscript. All authors read and approved this final version of the manuscript.

## Data Availability

1. Figshare. Coiling of echogenic perineural catheters with integral stylet: A proof-of-concept randomized control trial in a sciatic nerve block simulator_Data.xlsx. https://doi.org/10.6084/m9.figshare.26491585.v1 ^5^ This project contains the following underlying data:
• Coiling of echogenic perineural catheters with integral stylet: A proof-of-concept randomized control trial in a sciatic nerve block simulator_Data.xlsx (Data from 40 catheter coiling attempts in a sciatic nerve block simulator randomized for the two coiling methods described above: method A and method B.) Data are available under the terms of the Creative Commons Zero “No rights reserved” data waiver (CC0 1.0 Public domain dedication).
2. Figshare. Pilot study: Coiling of Echogenic Sciatic Nerve Perineural Catheters with Integral Stylet. https://doi.org/10.6084/m9.figshare.26854939.v1 ^6^ This project contains the following underlying data:
• Pilot study-original protocol.docx • Pilot study data.xlsx (data from 25 orthopaedic-trauma patients who had sustained tibia fractures and underwent a sciatic CPNB) • flow chart diagram.jpg • CONSORT Checklist.docx Data are available under the terms of the Creative Commons Zero “No rights reserved” data waiver (CC0 1.0 Public domain dedication). The pilot study conducted adheres to the CONSORT reporting guidelines. A copy of the original protocol, a completed CONSORT checklist, the pilot study’s data, as well as a flow diagram are available online through Figshare. https://doi.org/10.6084/m9.figshare.26854939.v1 ^6^
