# Supplementary material for: Coiling of echogenic perineural catheters with integral stylet: A proof-of-concept randomized control trial in a sciatic nerve block simulator and a pilot study in orthopaedic-trauma patients
Source: F1000Res. 2024 Dec 2;13:1103. Originally published 2024 Sep 27. [Version 2] doi: 10.12688/f1000research.155381.2 (PMC11617819; doi:10.12688/f1000research.155381.2)
Supplement: Peer review history

# Reviewer report: Reviewer response for version 2

**DOI:** 10.5256/f1000research.175369.r345084

**Published:** 2024-12-10

**Authors:** Agathi Karakosta

**Affiliations:** 1 University of Ioannina, Ioannina, Greece

I have read the revised manuscript carefully. I have no other comment to add.

Is the work clearly and accurately presented and does it cite the current literature?

Yes

If applicable, is the statistical analysis and its interpretation appropriate?

Yes

Are all the source data underlying the results available to ensure full reproducibility?

Yes

Is the study design appropriate and is the work technically sound?

Yes

Are the conclusions drawn adequately supported by the results?

Yes

Are sufficient details of methods and analysis provided to allow replication by others?

Yes

Reviewer Expertise:

Risk factor analysis, intraoperative haemodynamics, obstetric anaesthesia

I confirm that I have read this submission and believe that I have an appropriate level of expertise to confirm that it is of an acceptable scientific standard.

# Reviewer report: Reviewer response for version 2

**DOI:** 10.5256/f1000research.175369.r345083

**Published:** 2024-12-04

**Authors:** Konstantinos Kalimeris

**Affiliations:** 1 University of Zurich, Zurich, Switzerland

The authors have provided explanations and the appropriate corrections

Is the work clearly and accurately presented and does it cite the current literature?

Partly

If applicable, is the statistical analysis and its interpretation appropriate?

Partly

Are all the source data underlying the results available to ensure full reproducibility?

Yes

Is the study design appropriate and is the work technically sound?

Partly

Are the conclusions drawn adequately supported by the results?

Partly

Are sufficient details of methods and analysis provided to allow replication by others?

Partly

Reviewer Expertise:

Anesthesiology

I confirm that I have read this submission and believe that I have an appropriate level of expertise to confirm that it is of an acceptable scientific standard.

# Reviewer report: Reviewer response for version 1

**DOI:** 10.5256/f1000research.170554.r342696

**Published:** 2024-11-25

**Authors:** Konstantinos Kalimeris

**Affiliations:** 1 University of Zurich, Zurich, Switzerland

This paper presents an interesting method to coil perineural catheters which are not self-coiling. The authors conducted a study in Blue Phantom comparing if removing the stylet from the catheter before threading the catheter improves the coiling and then used this technique in 25 patients. They found that removing the stylet before threading the catheter improves coiling and they propose a solution for the obstruction of the catheter. 

 - The purpose of the study is not clearly stated. Is it to test a technique to coil a straight stiff catheter? 

 -  Introduction should better explain the background, which research question this study aims to answer and why is it interesting to know.

 For example: refer 1.

 This reference should be added.

 What is the benefit from not using self-coiling catheters? Nickl et al report obstruction at 1.3%. Is there a clear cost difference?

**- Methods:**

 Authors should state if the coiling of the catheter is reliably recognized by ultrasound in all patients and Phantom. Some better quality images could help the average reader to understand the technique.

 An important question that needs to be answered and stated in the paper is if this technique is according to the manufacturer's instructions or off-label. Does the technique  involve retracting the catheter while inside the needle? Would a shear or damage of the catheter be possible by the additional manipulations? 

 - The technique should be more clearly described. Was the coiled catheter placed inside the paraneurium? 

 Would coiling inside the paraneurium possibly cause problems when removing the catheter? (knotting?)

 Please state where/how many the openings of the catheter are.

 The 15sec time limit is confusing. Please explain more the meaning of the measurement or remove

 What do Authors mean that "the case was excluded" when the anesthetic didnt reach the nerve? Please state more clearly in how many cases the technique was not feasible, as the paper regards feasibility of a new technique.

 The  study is powered for comparing two methods in the Phantom with great difference, but not for dislocation or other endpoints in the patients. There is no control group. Results are interesting, but conclusions must be carefully drawn, especially regarding complications.

 A section with the above limitations of the study should be included in the Discussion section.

**Results/Discussion:**

 Figure 3 is unnecessary. Since the paper is a presentation of a technique, ultrasound images of the catheter threading and images where the coiling is clearly seen would be more helpful.

 It would add if authors included a short literature-based comparison to other techniques of prolonged regional anesthesia.

 From conclusion and abstract please remove "easily". Authors report 12% not possible placement and 8% obstruction. A comment on the safety of the technique should be added.

Is the work clearly and accurately presented and does it cite the current literature?

Partly

If applicable, is the statistical analysis and its interpretation appropriate?

Partly

Are all the source data underlying the results available to ensure full reproducibility?

Yes

Is the study design appropriate and is the work technically sound?

Partly

Are the conclusions drawn adequately supported by the results?

Partly

Are sufficient details of methods and analysis provided to allow replication by others?

Partly

Reviewer Expertise:

Anesthesiology

I confirm that I have read this submission and believe that I have an appropriate level of expertise to confirm that it is of an acceptable scientific standard, however I have significant reservations, as outlined above.

## References

[1] : Impact of self-coiling catheters for continuous popliteal sciatic block on postoperative pain level and dislocation rate: a randomized controlled trial. *BMC Anesthesiol* .2022;22(1) : 10.1186/s12871-022-01700-x 159 10.1186/s12871-022-01700-x 35610566 PMC9128226

# Response

**Published:** 2024-11-27

**Authors:** Eleni Poulogiannopoulou

**Affiliations:** 2nd Department of Anaesthesia, National and Kapodistrian University of Athens School of Medicine, Athens, Attica, Greece

We would like to thank you for the feedback and comments. In the revised manuscript, all recommendations were considered and incorporated in the new text.  Corrections in the text are highlighted with bold to facilitate perusal.

**1) The purpose of the study is not clearly stated. Is it to test a technique to coil a straight stiff catheter?**

 The catheter used in the present study was not stiff; it was not made of potentially rigid material (such as plastic) but the material of the applied perineural catheters was polyurethane based: This detail is added in the introduction section (paragraph 3: purpose of the study) and in the methods section as well (paragraph 2; page 4; line 3-5). Additionally, the second paragraph of the introduction section is redrafted, further elaborating on the purpose of the study. The reference of Nickl R et all is now cited in the reference list.

**2) What is the benefit from not using self-coiling catheters? Nickl et al report obstruction at 1.3%. Is there a clear cost difference?**

 A procedural flaw may be the kinking of the distal end of the coiled catheters. Although this is a major shortcoming of the technique, by partially uncoiling the catheters, points of obstruction were relieved (Discussion section; page 10; paragraph 4).

**3) Authors should state if the coiling of the catheter is reliably recognized by ultrasound in all patients and Phantom. Some better-quality images could help the average reader to understand the technique.**

 The coiling maneuver was clearly recognized both in the blue phantom material and the case series by the investigators. The coiling maneuver of the catheter was recognizable due to its dynamic coiling motion during advancement (frame by frame analysis of the catheter in the stored video loops). In fact, the interobserver agreement of coiling maneuver between investigators was significant (k index=90) (Figure 3A). A video clearly presenting the motion of the catheter and its coiling maneuver during advancement was added (data availability section).

**4) An important question that needs to be answered and stated in the paper is if this technique is according to the manufacturer’s instructions or off-label. Does the technique involve retracting the catheter while inside the needle? Would a shear or damage of the catheter be possible by the additional manipulations?**

 This technique did not follow the manufacturer’s instructions but is an off-label technique. In fact, the methodology to sufficiently evaluate needles and perineural catheters on patients (not following manufacturer’s instructions) presents difficulties and/or ethical concerns. Therefore, we set out to initially assess this specific perineural catheter method in a “human-tissue mimicking” simulation model (Discussion section; page 9; paragraph 2).

 In fact, if the catheter was not coiled with the first attempt, it was then gently retracted and re-advanced; nevertheless, neither shear nor obvious damage nor knotting of the catheter were noticed (Results section; page 8; paragraph 3).

**5) The technique should be more clearly described. Was the coiled catheter placed inside the paraneurium?**

**Would coiling inside the paraneurium possibly cause problems when removing the catheter? (knotting?)**

 The technique is more clearly described by incorporating in the text more details regarding the catheter placement in the paraneurium (Methods section; page 7; paragraph 2, 3 and 4)

 Knotting was not noticed in both studies.

**6) Please state where/how many the openings of the catheter are?**

 The catheter had one opening on its tip (Methods section; page 4)

**7) The study is powered for comparing two methods in the Phantom with great difference, but not for dislocation or other end points in the patients. There is not a control group. A section with the above limitation should be included.**

 Indeed, our clinical study was not powered, nor did it include a control group. This is an obvious methodology weakness, even though the intention was to deploy a pilot study supplementary to the simulation study, in addition to providing definitive preliminary data for a future randomize control clinical trial. We concur with the reviewer’s comment and a new paragraph in the limitation section was added.

**8) Figure 3 is unnecessary. Since the paper is a presentation of a technique, ultrasound images of the catheter threading and images where the coiling is clearly seen would be more helpful.**

 Figure 3 is omitted, and an explanatory video is included (data availability section).

**9) From conclusion and abstract please remove "easily". Authors report 12% not possible placement and 8% obstruction. A comment on the safety of the technique should be added**

 Both in the conclusion and abstract section the word "easily" was removed.

**10) It would add if authors included a short literature-based comparison to other techniques of prolonged regional anesthesia.**

 A new reference was added Nickl R et all in the reference list, emphasising the use of other types of coiled catheters in CPNB applications. In order to comply with the word limit (2500), we decided upon minimising the above comparison. Our article is a technical brief report focusing on specific technical details.

**11) The 15sec time limit is confusing. Please explain more the meaning of the measurement or remove**

 The 15 sec limit was an arbitrary decision, because we aimed to proceed with the simulation in a time limiting manner. In fact, the reviewer correctly considers that the certain time limit potentially confuses the implementation of the technique, but for the reason of group comparisons (coiling ability) a limited time period of implementation was imperative.

# Reviewer report: Reviewer response for version 1

**DOI:** 10.5256/f1000research.170554.r327786

**Published:** 2024-11-06

**Authors:** Agathi Karakosta

**Affiliations:** 1 University of Ioannina, Ioannina, Greece

**COMMENTS TO THE AUTHORS**

 First, I would like to congratulate the authors for the interesting and relevant work. Data is presented in a detailed and coherent manner.

 However, there are minor issues that need clarification.

 1) A flow chart for both the simulation and the pilot study would be helpful (depicting number of attempts, excluded cases or any other relevant detail).

 2) What statistical test did you employ for examining normal distribution of continuous variables. Please include this information in the “statistical analysis” section. Moreover, the way results are presented (i.e., mean(SD) or median(IQR) for continuous data and absolute/relative frequencies for categorical data or any other way ) should be mentioned in “statistical analysis”.

 3) In results you state “oxycodone consumption was 12.5 (0-20) mg …”. Are you referring to mean and range or median and IQR (interquartile range)? Please specify.

Is the work clearly and accurately presented and does it cite the current literature?

Yes

If applicable, is the statistical analysis and its interpretation appropriate?

Yes

Are all the source data underlying the results available to ensure full reproducibility?

Yes

Is the study design appropriate and is the work technically sound?

Yes

Are the conclusions drawn adequately supported by the results?

Yes

Are sufficient details of methods and analysis provided to allow replication by others?

Yes

Reviewer Expertise:

Risk factor analysis, intraoperative haemodynamics, obstetric anaesthesia

I confirm that I have read this submission and believe that I have an appropriate level of expertise to confirm that it is of an acceptable scientific standard.

# Response

**Published:** 2024-11-27

**Authors:** Eleni Poulogiannopoulou

**Affiliations:** 2nd Department of Anaesthesia, National and Kapodistrian University of Athens School of Medicine, Athens, Attica, Greece

Thank you very much, indeed, for all your significant remarks. In the revised manuscript, all recommendations were considered and incorporated in the new text.  Corrections in the text are highlighted with bold to facilitate perusal.

**1) A flow chart for both the simulation and the pilot study would be helpful (depicting number of attempts, excluded cases or any other relevant detail).**

 A flow chart is included in Figshare. Pilot study: Coiling of Echogenic Sciatic Nerve Perineural Catheters with Integral Stylet. https://doi.org/10.6084/m9.figshare.26854939.v1

**2) What statistical test did you employ for examining normal distribution of continuous variables. Please include this information in the “statistical analysis” section. Moreover, the way results are presented (i.e., mean(SD) or median(IQR) for continuous data and absolute/relative frequencies for categorical data or any other way ) should be mentioned in “statistical analysis”.**

 Normality was assessed by D'Agostino-Pearson test and the continuous data were described as mean (range) (Statistical analysis section).

**3) In results you state “oxycodone consumption was 12.5 (0-20) mg …”. Are you referring to mean and range or median and IQR (interquartile range)? Please specify.**

 Oxycodone consumption is presented as mean (range: highest value-lowest value).
